# Micro-/Nano-Carboxymethyl Cellulose as a Promising Biopolymer with Prospects in the Agriculture Sector: A Review

**DOI:** 10.3390/polym15020440

**Published:** 2023-01-13

**Authors:** Roohallah Saberi Riseh, Mozhgan Gholizadeh Vazvani, Mohadeseh Hassanisaadi, Yury A. Skorik

**Affiliations:** 1 Department of Plant Protection, Faculty of Agriculture, Vali-e-Asr University of Rafsanjan, Imam Khomeini Square, Rafsanjan 7718897111, Iran; 2 Department of Plant Protection, Faculty of Agriculture, Shahid Bahonar University of Kerman, Kerman 7618411764, Iran; 3 Institute of Macromolecular Compounds of the Russian Academy of Sciences, Bolshoi VO 31, St. Petersburg 199004, Russia

**Keywords:** carboxymethyl cellulose, superabsorbent hydrogels, active edible coatings, encapsulation, bacteria

## Abstract

The increase in the population rate has increased the demand for safe and quality food products. However, the current agricultural system faces many challenges in producing vegetables and fruits. Indiscriminate use of pesticides and fertilizers, deficiency of water resources, short shelf life of products postharvest, and nontargeted delivery of agrochemicals are the main challenges. In this regard, carboxymethyl cellulose (CMC) is one of the most promising materials in the agriculture sector for minimizing these challenges due to its mechanical strength, viscosity, wide availability, and edibility properties. CMC also has high water absorbency; therefore, it can be used for water deficiency (as superabsorbent hydrogels). Due to the many hydroxyl groups on its surface, this substance has high efficacy in removing pollutants, such as pesticides and heavy metals. Enriching CMC coatings with additional substances, such as antimicrobial, antibrowning, antioxidant, and antisoftening materials, can provide further novel formulations with unique advantages. In addition, the encapsulation of bioactive materials or pesticides provides a targeted delivery system. This review presents a comprehensive overview of the use of CMC in agriculture and its applications for preserving fruit and vegetable quality, remediating agricultural pollution, preserving water sources, and encapsulating bioactive molecules for targeted delivery.

## 1. Introduction

The development of industry and industrial activities has increased the levels of environmental pollution caused by the heavy use of nondegradable materials. Therefore, the production of degradable and environmentally compatible materials should be considered an important direction in industrial activities. Similarly, the world’s population is expanding daily, and the United Nations now predicts that the world population in 2050 will be 9.7 billion people [1]. Therefore, research should focus special attention on agriculture and meeting the food needs of this population. The quantity and quality of food can be improved by adapting ideas from drug delivery systems and nanotechnology for use in the agricultural sector [2]. However, many of the materials used in drug delivery and nanotechnology are derived from synthetic polymers produced from petroleum and coal and are incompatible with agricultural products destined for consumption. They are also incompatible with the environment, as they do not undergo natural recycling.

By contrast, biological materials, such as starch, cellulose, chitin, chitosan, zein, and gelatin, by virtue of their adaptability, durability, and price, could gradually replace synthetic polymers to resolve the problems inherent in synthetic materials [3,4,5]. One versatile material is carboxymethyl cellulose (CMC), a cellulose derivative that is widely used in industry. CMC is a linear polysaccharide of anhydro-glucose connected as repeating units joined by β-1,4-glycosidic bonds. This composition has mechanical strength, tunable hydrophilicity, viscosity, and abundant availability. CMC is widely used in the food, paper, textile, and pharmaceutical industries, as well as in biomedical engineering, wastewater treatment, energy production, and maintaining the quality of agricultural products [6]. Of the several naturally occurring polysaccharides, cellulose and chitin are the most important biopolymers that have been put to many uses in biomedical fields (tissue engineering, wound healing, and drug delivery). The advances made have expanded to the use of the nanotechnology and biopolymers in the food [7,8,9], pharmaceutical [10,11], and agricultural [12] industries. The size of particles distinguishes nano-CMC from micro-CMC. The size of nano-CMC ranges from 5–100 nm, while micro-CMC ranges from 100–200 μm. When the size is down to the nanoscale, the high specific surface area of nano-CMC increases the efficiency [13].

Nano-based formulations have many applications in pesticide and nutrient delivery, and many other agricultural activities. For use in the field, these formulations should have high stability in outdoor conditions (sun, heat, and rain) while also retaining good solubility, dispersion, stability, mobility, and targeted delivery characteristics [14]. Many studies have documented the potential of nano-/micro-CMC-based formulations for the encapsulation and targeted delivery of biological control agents (BCAs) [15,16,17]. For example, encapsulation in micro-CMC and targeted application has been shown to extend the shelf life of bioactive compounds while reducing the required number of applications and concentration [18].

**Figure 1:**
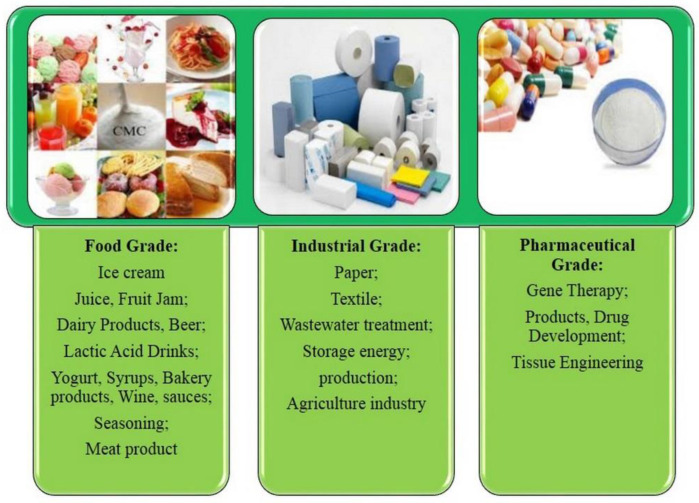
Applications of carboxymethylcellulose (CMC) in different industries.

At present, research has revealed that CMC is effective in the controlled release of agrochemicals. The relevant characteristics of nanomaterials, such as intelligent controlled-release properties, efficiency (high potential and productivity of CMC depending on the target), and environmental friendliness, can be exploited in the design of intelligent formulations for pesticide delivery [14,19,20]. For example, the controlled release of an herbicide, such as acetochlor, based on a diffusion mechanism [21] improves the soil-retaining capacity and urea leaching loss rate of sandy soil [22,23]. The effectiveness of the application of CMC depends on its purity, degree of polymerization (DP), degree of substitution (DS), and uniformity [6]. CMC products are currently used in the three different categories shown in Figure 1.

These polymers feature a number of useful properties, such as responsiveness to pH, time, temperature, chemical species, and biological conditions [24]. The many uses of hydrogels in different industries make them promising options for agriculture. Several scientists have recently shown that CMC has the potential to serve as an active material in the remediation of heavy metals (HMs) and chemical pesticides [25,26]. In addition, hydrogels based on CMC can be used as superabsorbents in agriculture due to their ability to absorb and retain water [27]. Coating fruits and vegetables with edible polymers such as CMC can provide a protective barrier against physical, biochemical, and microbial damage and can substitute for conventional preserving methods.

The potential of CMC-based formulations indicates that this biopolymer could be a safe substance for agricultural applications aimed at the formulation of BCAs, remediation of pesticides and HMs, water retention under drought stress, and protection of agricultural products from different types of damage. This literature review presents an extensive overview of the use of CMC in agriculture, including the preservation of the quality of fruits and vegetables, remediation of agricultural pollution, preservation of water sources, encapsulation of bioactive molecules, and targeted delivery of those molecules.

In Table 1, a summary of CMC applications is given, which will be explained in detail later.

## 2. Cellulose and Carboxymethyl Cellulose and Their Properties

**Figure 2:**
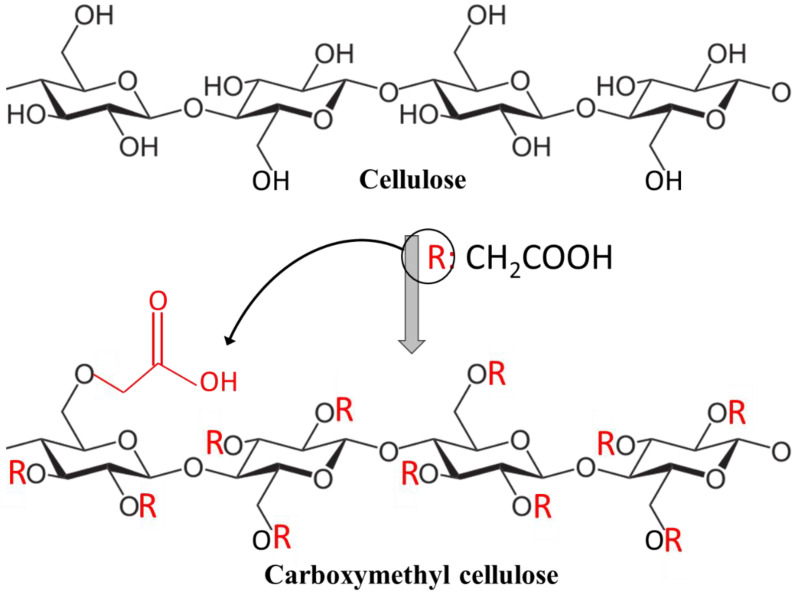
Chemical structure of cellulose and carboxymethyl cellulose.

Among the naturally occurring biopolymers, cellulose is the most abundant, with large quantities found in the plant cell walls, algae, and oomycetes (in the form of microfibrils). Cellulose is a linear homopolysaccharide composed of β–1,4-linked D-glucopyranose units (Figure 2), which can be converted into valuable cellulose esters and ethers [37,38,39].

Cellulose is insoluble in water [40,41]. Its extensive occurrence in nature makes it an inexhaustible resource, so this polymer can be used to produce useful products. Various sources contain cellulose, including used bedding, straw, rice husks, waste wood, and sunflower waste. The sources can range from wood to agricultural waste and can be viewed as inexhaustible resources for industrial activities [42,43].

**Figure 3:**
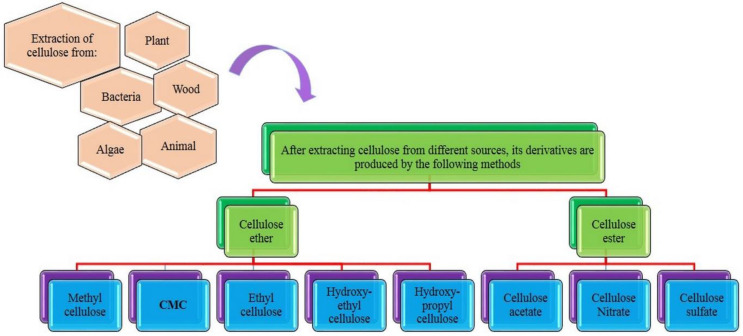
Different sources of cellulose and types of derivatives of this polymer.

Cellulose neither melts nor dissolves readily in hot or cold water solvents due to its strong inter- and intramolecular hydrogen bonds to nearby oxygen [44]. It has other undesirable properties, such as moisture sensitivity and low resistance to microbial attacks [44,45,46]. Therefore, this polymer should ideally be converted into more amenable derivatives. The sources of cellulose, its derivatives, and the methods of producing its derivatives are shown in Figure 3.

CMC is one of the most important cellulose derivatives. This polymer, due to its characteristic properties, such as mechanical strength, tunable hydrophilicity, viscous properties, and low-cost synthesis process, as well as the availability and abundance of raw materials, is now widely used in various advanced application fields [6]. The unique chemical structure of CMC is the presence of -CH_2_-COOH groups attached to some of the hydroxyl groups of the cellulose units. This chemical structure makes CMC soluble in water, unlike cellulose. In addition to its water solubility, it has a high viscosity and moderate strength, and is odorless, tasteless, nontoxic, nonallergenic, flexible, transparent, and resistant to oil and fats, moisture, and oxygen transmission [47,48,49,50]. Figure 2 depicts the chemical structure of CMC.

The synthesis of CMC from its various conventional (plant-based) and nonconventional (waste materials) precursors and their essential characterization techniques, such as scanning electron microscopy, Fourier transform infrared spectroscopy, and thermogravimetric analysis, will be discussed extensively in later sections [6]. Residues of agricultural products, such as straw and stubble left over from crop harvests, contain large amounts of cellulose that can be used for specific economic and environmental benefits [51]. Plant-based precursor materials used as the source of CMC are the following: (1) waste materials and (2) nonwaste materials. Agricultural wastes that contain high amounts of cellulose include maize stalks, cacao pod husks, the pulp of *Eucalyptus globulus*, orange peel, sugarcane bagasse, *Asparagus officinalis* stalk ends, rice straw, wheat straw, coconut fibers, corn cobs, cotton ginning trash, cotton linters, dried duckweed, durian fruit rind, fig stems, *Mimosa pigra* peel, mulberry paper, oil palm fibers, palm bunches, palm kernel, papaya peel, pineapple peel, rice hull, rice stubble, sago pulp, sugar beet pulp, and sugarcane straw [52]. The synthesis of CMC has been reported from paper sludge, wood residue, textile wastes, mixed office waste, and terry towel waste [53,54,55]. Table 2 depicts differences between cellulose and CMC.

## 3. Synthesis and Characterization of CMC

CMC is an anionic and water-soluble linear polymer. This compound is ionic in nature. Due to its ionic properties, this compound is sensitive to the presence of most electrically charged molecules. This compound is odorless and clear and forms a clear solution. Furthermore, CMC coatings have some other desirable characteristics, including: perfect water solubility, tastelessness, odorless, high viscosity, non-toxicity, moderate strength, transparency, flexibility, resistance to fats and oils, moderate moisture, and oxygen transmission [56].

Hydrogels such as CMC are useful for absorption due to the presence of hydrophilic polymer networks and are prepared through chemical or physical cross-linking. In physical methods, molecular chains are held together by intermolecular forces such as molecular entanglements, electrostatic interactions, and H-bonding so that they are easily distributed. In chemical methods, chains are cross-linked by cross-linking agents, resulting in more stable hydrogels than those prepared by physical methods [57].

**Figure 4:**
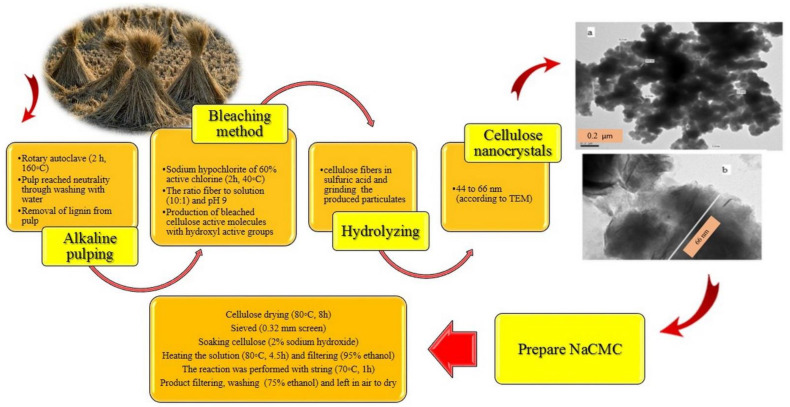
Cellulose production from rice straw and preparation of NaCMC.

Different methods for the synthesis of CMC have been reported in different studies. For example, cellulose obtained from rice straw with an alkaline pulping process was used to make CMC nanocellulose in a tramadol drug-loaded capsule [51]. Generally, the synthesis of CMC includes alkalization and etherification, as explained in this section. However, in brief, in the alkalization and etherification process, firstly cellulose is alkalized with sodium hydroxides (NaOH), then the CMC from is synthesized through the etherification of cellulose using sodium monochloroacetic acid [58]. A summary of the synthesis and preparation of NaCMC [51] is shown in Figure 4.

In other research, cellulose was first extracted from asparagus stalks and stored in a polyethylene glycol bag. Different concentrations of NaOH (20, 30, 40, 50, and 60) and 350 mL of isopropanol were blended with 15 g of cellulose powder. The etherification reaction solution was heated (45–75 °C, 1 h) and then filtered. The filtrate was suspended in methanol (100 mL) and neutralized with acetic acid (10 mL) to form a yellowish solid product. The solid product was washed with ethanol (5 times, 50 mL), followed by a one-time wash with absolute methanol to remove sodium glycolate and chloride, and then dried (temperature: 60 °C, time: 3 h) [59,60]. After determining the desired source for cellulose extraction and CMC synthesis, calculation of the degree of CMC substitution is required. The degree of substitution (DS) of CMC (Equation (1)) means the average number of hydroxyl groups of cellulose substituted with carboxymethyl and sodium carboxymethyl groups [59]. The yield of CMC was calculated using Equation (2) [59,60,61].

(1) $$DS=\frac{1150M\;\mathrm{content}}{(7120-412M-80C)\mathrm{Content}}+\frac{(162+58A)C\;\mathrm{content}}{(7120-80C)\mathrm{content}}$$

(2) $$\mathrm{Yield}\;\mathrm{of}\;\mathrm{CMC}\;(\mathrm{wt.}\%)=\frac{\mathrm{Weight}\;\mathrm{of}\;\mathrm{dried}\;\mathrm{CMC}\;(g)}{\mathrm{Weight}\;\mathrm{of}\;\mathrm{dried}\;\mathrm{cellulose}\;(g)}$$

The purity of CMC can be determined using the method described by Golbaghi, Khamforoush, and Hatami Golbaghi et al. [62]. Two grams of CMC precipitate was added to distilled water (50 mL, 80 °C) and dissolved. The solution was centrifuged (4000 rpm) and the solid precipitate was removed. The solution was mixed with pure acetone (50 mL) to recover the CMC. The CMC obtained was filtered, dried at 70 °C, and its purity determined using Equation (3) [59,60,61].

(3) $$\mathrm{Purity}\;(\%)=\frac{\mathrm{Weight}\;\mathrm{of}\;\mathrm{dried}\;\mathrm{residue}}{\mathrm{Weight}\;\mathrm{of}\;\mathrm{specimen}\;\mathrm{used}}\times 100$$

Several parameters related to CMC should be checked to characterize CMC; these include: (1) morphology analysis by scanning electron microscopy (SEM), (2) confirmation of the presence of functional groups with FT-IR, (3) recording of XRD patterns of the products by X-ray diffractometry, and (4) determination of solubility and pH of CMC by adding 5 g of powder to 50 mL of double distilled water [59,60,61]. In one study, 15 g of oil palm frond cellulose powder was alkalized (at 30 °C for 60 min in a water bath shaker) with 50 mL of 37.9%, 40%, 45%, 50%, or 52.1% NaOH in 450 mL isopropanol. Upon completion of the alkalization process, the etherification process was initiated by adding various amounts of monochloroacetic acid (10.7, 12, 15, 18, and 19.2 g) at 50 °C for 3 h. After filtration, the slurry was suspended in methanol and neutralized with 90% acetic acid. The resulting powder was designated as the CMC product. Byproducts were removed by washing the powder four times with 80% ethanol. The yield of CMC was calculated based on the amount of cellulose. The cellulose and CMC morphology was analyzed by SEM. The moisture content, degree of substitution (DS), and purity of CMC were determined. The substitution reaction was confirmed by the presence of the COO-, -CH_2_, and -O- groups in the IR spectrum. The results showed a rough exterior surface of cellulose with twisted and ruptured fibers (due to the use of strong chemicals and a high temperature in the cellulose extraction process). However, the synthesized CMC showed decreased roughness (due to a change in cellulose crystallinity) [63]. In another study, cotton samples were prepared with different degrees of polymerization and used to synthesize CMC. The alkaline treatment was performed with sodium hydroxide and boiling water. The perborate treatment was performed with sodium perborate, urea, nonionic wetting agent, and cold water. The carboxymethylation reaction was performed with isopropyl alcohol, NaOH, Na_2_CO_3_, methanol, and acetic acid. The degree of substitution (DS) of CMC was calculated, and the carboxyl content of CMC was measured. Silver nanoparticles (AgNPs) prepared using this CMC were useful as antimicrobial agents for textiles [64].

A number of studies that prepared CMC by different methods are given in Table 3. The general methods for CMC synthesis are similar in most cases, with differences only in the number of materials used, temperature, and time. CMC synthesis methods from different cellulose sources, including almond shells, papaya peel, *Mimosa pigra* peel, sugar beet pulp, Cavendish banana pseudostem, and sago waste, have been reported [60,65,66,67,68,69]. 

## 4. CMC Applications in Agriculture

CMC is widely used in industry because of its capacity to act as a water-retaining, emulsifying, or film-forming agent, and because it is nontoxic, renewable, inexpensive, hydrophilic, and biodegradable [73]. These features promote its use as a carrier molecule with different characteristics, depending on the purpose. Global climate change and the decreases in annual rainfall across the globe [52,74], as well as various pests and diseases that cause significant damage to agricultural products every year, have directed special attention to cellulose-based hydrogels. In the agricultural sector, the use of nanotechnology to produce pesticides to create new formulations has led to the development of efficient and safe pesticides. The purpose of developing these nanomaterials is to allow the release of sufficient amounts of their active substances in response to environmental stimuli and biological needs through controlled-release mechanisms [14]. Various polymers have been used as carriers for agricultural inputs (fertilizers and pesticides), pharmaceuticals, and dietary supplements. Hydrogels are ideal for improving water efficiency in water-stress agriculture [75,76]. The availability of water and fertilizers influences agricultural production. Furthermore, the increase in fertilizer costs has had a direct impact on the recent rise in food prices, so fertilizers with lower costs and higher efficiency need attention [76,77]. Targeted fertilizer delivery systems with a high delivery capability, controlled release of nutrients, control of pests and diseases, and increasing resistance to drought are special goals that should be considered in the agricultural sector.

### 4.1. CMC in the Targeted Delivery System

The significant progress made recently in materials science, nanotechnology, and hydrogels has led to the suggested use of advanced delivery systems in different fields of agriculture [78,79,80]. Nutrition management plays a very important role in the high performance of plants. If the nutrients needed by the plant are available in sufficient quantities, its defense system will overcome many pests and diseases. Drought is another limiting factor that can endanger crop production, performance, and quality. Therefore, improving the utilization of water resources and fertilizer nutrients is important, especially for urea, which is the most widely used fertilizer and has a high nitrogen content (46%). This fertilizer is absorbed by the plant in only small amounts and cannot be easily adsorbed to the charged particles of the soil. For this reason, irrigation washes excess fertilizer from the surface of agricultural land and causes environmental pollution [81].

One way to overcome these current limitations of fertilizer use is to use controlled-release fertilizers, which have several advantages, including a lower fertilizer loss rate, sustainable nutrient supply, lower application frequency, and minimal potential negative effects associated with overdosage. Superabsorbent polymers are promising for use as water- and nutrient-managing agents in arid and desert areas [81]. Polymer coatings are the most common mechanism of the controlled release of chemical fertilizers. The polymer coating slowly decomposes during the growing season, and the fertilizer is gradually provided to the plant. Coating fertilizers with a polymer such as CMC helps immobilize the fertilizer particles, making them resistant to runoff and leaching [82].

Mohamed, Fahim, and Soliman [76] recently synthesized CMC (0.25 g/L) and poly 4-vinylpyridine (P4VP) hydrogel systems with different ratios in the presence of cross-linker N,N’-methylene bisacrylamide and investigated their biodegradability in soil for future large-scale use in agriculture. The hydrogel characteristics were characterized by FTIR spectroscopy, thermal gravimetric analysis, X-ray diffraction, and scanning electron microscopy. The hydrogel yield, swelling ratio (SR) under different pH values, biodegradability of the hydrogel, loading capacity of the CMC/P4VP hydrogel, and fertilizer (calcium nitrate and urea) release from CMC/P4-VP hydrogel were calculated according to the following formulas:

Hydrogel extent of degradation was monitored by calculating the weight loss (Wt_loss_) according to Equation (4):

(4) $${\mathrm{Wt}}_{\mathrm{loss}}=[(\frac{{\mathrm{wt}}_{i}-{\mathrm{wt}}_{f}}{{\mathrm{wt}}_{i}})\times 100]$$

wt_i_ is the initial weight of samples before starting the degradation;
wt_f_ is the weight of the sample after specified time intervals of biodegradation.

The loading capacity of the hydrogels was calculated using Equation (5) with different fertilizer ratio:

(5) $$\mathrm{Fertilizer}\;\mathrm{loading}=[(\frac{{\mathrm{wt}}_{i}-{\mathrm{wt}}_{0}}{{\mathrm{wt}}_{0}})\times 100]$$

Wt_f_ is the weight of the loading hydrogel;
Wt_0_ is the weight of the unloaded hydrogel

Hydrogel yield was calculated according to Equation (6):

(6) $$\mathrm{Hydrogel}\;\mathrm{yield}=[(\frac{{\mathrm{wt}}_{H}}{{\mathrm{wt}}_{i}})\times 100]$$

wt_H_ is the final weight of dried hydrogel;
wt_i_ is the initial weights of CMC and P4VP.

The fertilizer release from the hydrogel after loading on CMC/P4VP was investigated by atomic absorption spectrometry under the following conditions: 8 mL buffer solution, pH 8, temperature = 20.4°C, and relative humidity = 47.6%. The urea loaded onto the hydrogel filled the hydrogel pores. The swelling ratio (SR) result confirmed a high swelling capacity (1100–1420%) for the CMC/P4VP hydrogel. The FTIR results indicated a strong interaction between the CMC fertilizer and P4VP fertilizer.

Biodegradation of the polymer is a desired feature in the environment and agriculture. In this experiment, the hydrogel lost 50% of its weight on day five. The structure of the CMC:P4VP:Urea with a l:1:1 ratio showed the highest loading capacity (62%) compared to the CMC:P4VP:Ca(NO_3_)_2_ with a 1:1:1 ratio (35%). The formation of the H-bond between CMC and urea appeared to enhance the urea absorption. Fertilizer release was examined using different mathematical models (e.g., zero order, first order, Korsmeyer–Peppas, and Higuchi models). The release mechanism was characterized as Super Case II transport, which is strongly affected by pH changes [76]. These findings point to the importance of using CMC (2%)-based hydrogels as agricultural inputs. Previous studies have shown that vermiculite is not suitable for agricultural applications because it relies on a diffusion mechanism. By contrast, NPK-filled hydrogels can show a controlled release of nutrients [29].

**Figure 5:**
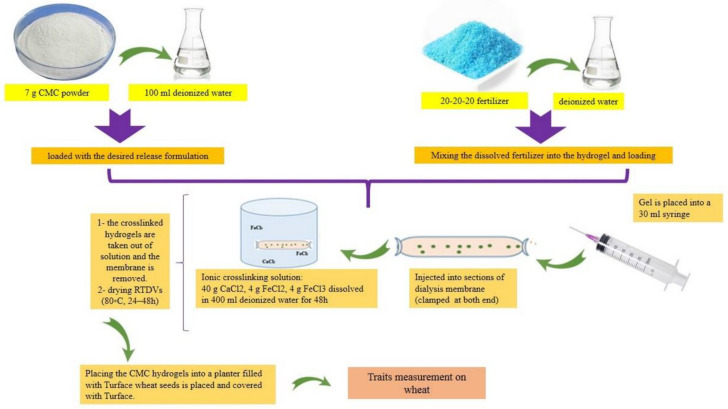
Root-targeted delivery vehicle (RTDV) synthesis as a targeted delivery system.

A CMC (20%, 40%, 50%, 60%, and 80%)-PVP hydrogel produced (thickness: 2 mm) in one study [29] was allowed to swell in water overnight and then at room temperature. The samples were weighed at different time intervals, and the moisture retention capability was measured. Urea was loaded by immersing dry CMC-PVP hydrogels into urea solutions of different concentrations (time: 24 h), and the swollen gels were dried (room temperature, 3 days). The samples retained about 50 wt.% of their water content (after 24 h, 25 °C) and showed an increased release of urea, suggesting that CMC/PVP superabsorbent hydrogels may be used as potential eco-friendly water-saving materials for agricultural applications [29]. Other research has used a root-targeted delivery vehicle (RTDV) in wheat. This system was formed by dissolving CMC (7%) chains in water, mixing them with liquid fertilizer, and then cross-linking using iron and calcium salts [30]. A summary of RTDV synthesis as a targeted delivery system is shown in Figure 5. 

This research showed that the tillers grew much taller in wheat supplied with the RTDV than in plants given daily fertilizer. The RTDV appeared to create conditions for the development of grains on the plant and increased the number of seeds, and significantly improved the seed yield. RTDVs were effective at increasing the growth and yield of wheat plants [30]. A dual-functional redox-responsive hydrogel was synthesized in other research [31]. This hydrogel was designed for two purposes: (1) the controlled release of agrochemicals and (2) the synchronous capture of heavy metal ions. CMC was used to construct a responsive matrix with cystamine as the linker. The redox reaction was carried out with the aim of in vivo drug delivery and organic synthesis through the exchange reaction of disulfide bonds [31]. These hydrogels were used in paddy soil contaminated with Cu^2+^ and Hg^2+^. After release, the hydrogels could capture heavy metal ions in soil via strong complexation (ion-thiol groups) disconnected disulfide bonds that could benefit plant growth and soil remediation [31]. However, although CMC has advantages in various industries, it also has limitations. Increasing the fertilizer concentration inside the hydrogel will also increase the release of the initial burst release of the fertilizer, making the overall release less stable. In addition, the presence of many ionic compounds, such as nitrogen, phosphorous, and potassium, in chemical fertilizers weakens the bond strength of CMC transverse joints and destroys their integrity. Therefore, a lower fertilizer concentration will increase the release duration and stability. Furthermore, soil moisture and climate change are some of the factors that can lead to inconsistencies in a controlled release, which may cause plants to be starved of nutrients or unable to use the released fertilizer [83].The initial release of fertilizer from hydrogel accounts for up to 90% in the first 80 h (approximately 3 days) [30]. However, this high release from hydrogels may not be ideal for agriculture. Plants need to have nutritional elements at their disposal and absorb them gradually and continuously. This especially applies to crops with a long growing season, such as wheat, barley, and alfalfa. In addition, the delivery of a high concentration of nutrients in the early stages of plant growth (such as germination), which occurs following sudden release (due to increased concentration), can harm the plant [30].

CMC-based hydrogels can be coated with more stable and resistant materials to withstand adverse conditions for long periods to achieve maximum efficiency. Cross-linking with metal ions, radiation, or with other naturally derived polymers is important in CMC (20%) hydrogels [84]. CMC derived from natural materials can hold much water in its structure and be used as a slow-release fertilizer [85]. Studies have shown that hydrogels prepared by cross-linking with a natural polymer show good biodegradability and mild and controllable reaction conditions, indicating that this is the most ideal method for the synthesis of CMC gels [84,86,87,88]. The research showed that adding fiber improved the tensile strength of CMC-sodium alginate-glycerol composite films [89]. Thickeners such as starch, sodium alginate, or gelatin can improve the mechanical properties of CMC films [90]. In a study, it was found that the combination of carboxyl groups of CMC-aldehydes and different isocyanides, led to the formation of a wide range of derivatives of CMC, which increased the efficiency of CMC and was an environmentally friendly process [91].

In a study, a novel superhydrophobic sodium carboxymethyl cellulose (CMC) modified polyurethane (MPU) as a membrane material for controlled release fertilizer (CRF) by the cross-polymerization of 4,4’-diphenylmethane diisocyanate (MDI) and poly CMC-based modified PU (CMC) was fabricated. The results showed that polyurethane-coated fertilizer and modified polyurethane-coated fertilizer coatings were prepared by attaching hydroxyl groups to isocyanate to form a carbamate. Modified polyurethane had a lower water absorption rate than polyurethane, and the modified polyurethane-coated fertilizer coating showed excellent hydrophobicity. The modified polyurethane-coated fertilizer coating surface is much smoother and flatter than the polyurethane-coated fertilizer coating surface. The lifetime of nitrogen release in the modified polyurethane-coated fertilizer increased to 140 days [92]. CMC and quaternary chitosan (QCS) synthesized for use as an emulsion delivery system had good long-term stability and can be used for drug delivery and in food production [84,93].

### 4.2. CMC for Encapsulation of Bioactive Materials

A nanopesticide formulation has many advantages, such as a remarkable targeted delivery, intelligent controlled-release properties, pronounced efficiency, and environmental friendliness [12]. Nanocarriers and nanotechnology can protect pesticides from high temperatures and radiation, thereby significantly improving their chemical stability [94,95]. A CMC-zein-based nanopesticide delivery system was used to improve adhesion and antiultraviolet properties [20]. Zein (from maize residues) is a safe protein with unique solubility, biocompatibility, biodegradability, antioxidation, and nontoxicity properties, and it can be used as a drug carrier in the food, cosmetic, and medical fields [20,96,97]. This protein, in the form of nanoparticles (NPs), has the potential to release bioactive molecules when used as natural delivery systems and following encapsulation [98]. Hao, Lin, Lian, Chen, Zhou, Chen, Xu, and Zhou [20] considered the following options: (1) CMC-g-PDMDAAC was formed by the graft copolymerization of sodium-CMC and DMDAAC as functional monomers, (2) Avermectin (AVM) was selected as a model pesticide, and (3) an AVM@P-Zein/CMC-g-PDMDAAC nanopesticide was prepared by electrostatic interaction encapsulation. In this study, FTIR, TGA, SEM, and particle size analyzers were utilized to characterize the structure of CMC-g-PDMDAAC and AVM@P-zein/CMC-g-PDMDAAC. Investigation of the encapsulation efficiency, antiUV light performance, and sustained-release rate of the pesticides confirmed that CMC-g-PDMDAAC encapsulated in AVM@P-Zein lowered the sustained-release rate of the pesticides (at 300 h, the release rate had slowed down by 10%) [20].

CMC can also be used to improve the performance of phosphorylated-zein-based nanopesticides. The stability, ultraviolet resistance, and adhesion capacity are important components determining the performance of phosphorylated-zein-based nanopesticides. The chromogenic (carboxyl and hydroxyl) groups of CMCs can absorb part of the ultraviolet light and have certain antiultraviolet effects [99]. Research using a freeze-drying technique encapsulated bioactive crude ethanolic extracts of *Eucalyptus camaldulensis* with alginate-CMC by mixing a sodium alginate solution–CMC solution (in a ratio of 2% to 1%) and adding 1 mL of varying amounts (50, 500, and 1000 mg) of ethanolic leaf extract of *E. camaldulensis* (stirred for 20 min). This mixture was added to a 3% CaCl·2H_2_O solution at a flow rate of 1 mL/min under high-speed homogenization (15,000 rpm, 10 min). The mixture was stirred for 60 min and centrifuged at 10,000 rpm for 30 min. A high percentage yield (from 70.4% to 81.5%) was obtained, demonstrating minimal material loss and the highest encapsulation efficiency [32]. The efficacy of these microcapsules against bacterial pathogens, such as *Bacillus cereus*, *Listeria monocytogenes*, and *Staphylococcus aureus,* showed that *Eucalyptus camaldulensis* encapsulated with alginate-CMC had good antimicrobial activities and could be used as a disease control agent with excellent encapsulation efficiency and a high percentage yield. These microcapsules also showed a high swelling index, but it was reduced as the extract concentration increased. The swelling index indicated the hydrophilic nature of the alginate and the hydrocolloidal properties of the CMC. These capsules were cytocompatible with human colon cells [32].

NPK fertilizer was also encapsulated in CMC-based nanocomposite acrylic acid (AA) in the presence of polyvinylpyrrolidone (PVP) and silica nanoparticles for water retention in the soil [27]. The FTIR results confirmed the successful encapsulation of the NPK fertilizer compound within the superabsorbent. Investigation of the hydrogel using scanning electron microscopy revealed high porosity. This porous network has advantages, as it increases the contact surface area of the hydrogel nanocomposite, allows easier and faster diffusion of water molecules into the hydrogel, and promotes water absorption. The water absorption capacity of hydrogels is significantly influenced by the salinity and pH of the swelling medium (the water content decreases in saline solutions and at high pH). These observations showed that a hydrogel/PVP/silica/NPK formulation could improve the utilization of fertilizers in agricultural and horticultural applications [27]. Table 4 summarizes studies on the CMC encapsulation of bioactive materials and targeted delivery systems in agriculture and other disciplines.

A change in the acidity of the environment is one factor that can affect CMC encapsulation performance. Acidic and alkaline soils each have a different effect on the encapsulation efficiency. Zein encapsulated by CMC as a pesticide showed different encapsulation efficiencies under acidic and basic conditions. The particle size and negative charge increased in response to pH changes. Increasing acidity caused carboxylate groups to undergo deprotonation and increased the negative charges on the capsule surfaces. The encapsulation efficiency and particle size at pH 9 were 80.18% and 304.17 nm (the highest value), respectively. At a low pH, the particle size became smaller, resulting in a shorter release route and a faster release rate [20].

Increases and decreases in rainfall can change the soil pH, which will change the encapsulation efficiency. Different tillage systems and microorganisms in the soil can also affect the pH. Another limiting factor controlling the release from microcapsules is temperature. The release of polyphenols is lower at a low temperature (4 °C) than at body temperature (37 °C) [32]. These cases demonstrate the factors that can limit the use of CMC in the form of microcapsules in agriculture and point to the need to use intermediate materials for the preparation of microcapsules to maintain the beneficial properties of CMC.

### 4.3. CMC as Superabsorbent Hydrogels

**Figure 6:**
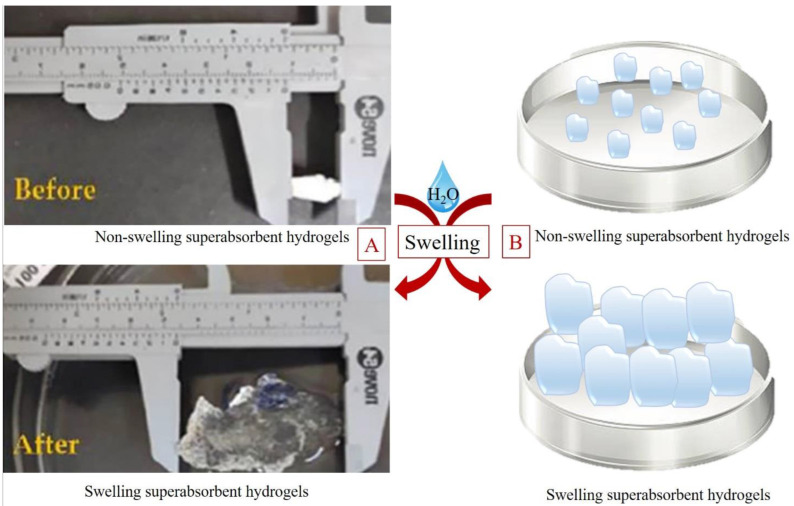
Swelling behavior of superabsorbent hydrogels (SAH) in a real sample (**A**) [106] and schematically (**B**).

In many countries, precipitation is confined to a few seasons per year and is insufficient for agricultural purposes at other times. Additionally, equipment such as drip irrigation systems is not amenable to home applications, and plants suffer from water stress during long journeys. In traditional agricultural systems, many plants are irrigated through traditional methods, such as flood irrigation, which makes large quantities of water accessible to the plant once at a certain time but reduces water consumption efficiency. This irrigation also results in much water absorption by weeds, which is a problem in agriculture. Therefore, a product that absorbs and retains a substantial amount of water with swelling behavior and permits the slow release of water would be highly advantageous. Figure 6 shows the swelling behavior of superabsorbent hydrogels. These products are important in modern agriculture because of their potential to modify soil permeability and evaporation rates.

**Figure 7:**
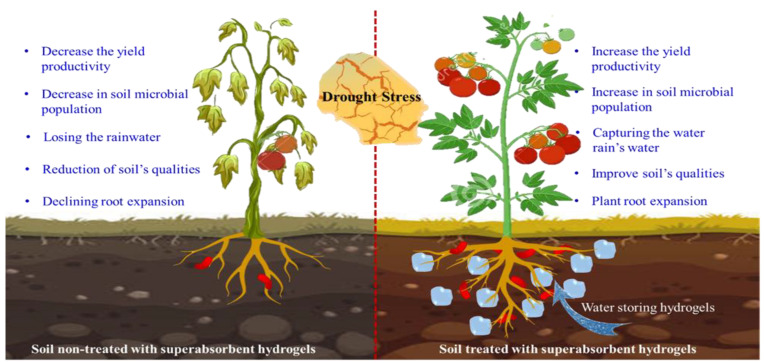
The advantages of using superabsorbent hydrogels (SAHs) for water holding in agriculture.

Superabsorbent hydrogels (SAHs) are 3D networks and hydrophilic structures with a high capacity for water absorption. By expanding, they can take up and hold amounts of aqueous solutions equivalent to up to 95% of their initial dry weight [107,108]. SAHs slowly release water into the soil and maintain soil moisture longer under water-stress conditions, thereby allowing plants to thrive through prolonged periods of water scarcity, increasing the yield productivity [109]. Li et al. [110] utilized SAHs as a soil additive and compared their effects on the water content, soil microbial populations, and crop production of *Triticum aestivum*. They found that incorporating SAHs into the soil had no observable negative consequences and improved both the soil physical qualities and crop productivity. Therefore, in times of drought, SAHs can be used as a private subsurface reservoir, capturing all the water that would normally be lost to evaporation (such as rainwater) and allowing continued agricultural production [109]. Figure 7 shows the agricultural advantages of SAHs for water holding.

With the aim of conserving water and enhancing crop productivity, SAHs based on cellulose are increasingly being employed in agricultural applications because of their many desirable characteristics, including their high hydrophilicity, biocompatibility, permeability, and high swelling ratio [111,112]. CMC, among the other cellulose derivatives (e.g., hydroxypropyl methylcellulose, hydroxyethyl cellulose, and methyl cellulose), is more amenable for preparing SAHs due to its high water absorbency and swelling ratio in distilled water and salt solutions [113]. One gram of 10% CMC-based SAHs can absorb approximately 450 g of water [113]. However, this ratio can change if CMC is combined with other materials. Indeed, in addition to the simplest CMC-based SAHs, some CMC-based SAHs have been fabricated to incorporate other chemical compounds that mainly play the role of cross-linkers. Feket et al. [113] produced SAHs containing 70% CMC and 30% starch and found a maximum water absorption of 350 g per g of SAHs. Salleh et al. [114] produced SAHs by combining cellulose from oil palm branches and sodium-CMC and using epichlorohydrin as a cross-linker to fabricate SAHs.

Other research has confirmed the revolutionary impact of cellulose-based SAHs in modern agricultural science. Cannazze et al. [115] revealed that soil amended with cellulose-based SAHs could conserve water and slowly release it to plant roots in times of drought. Therefore, these compounds are solutions that promise to solve the water shortage problem in the agricultural sector. Li et al. [116] claimed that SAHs also affect the soil microbial population under water deficit. Under water scarcity conditions, they revealed that SAH-treated soil had a high bacterial richness. Satriani et al. [117] applied a superabsorbent cellulose biopolymer in an irrigation system and reported an enhancement in the crop water productivity index. Based on the reviewed documents, SAHs appear to be practical substances for overcoming the problem of drought and water scarcity in agriculture. Table 5 shows some studies on the application of CMC as a superabsorbent hydrogel in agricultural activities.

### 4.4. CMC to Remediate Pesticides and Heavy Metals from Agricultural Water

Water is the world’s most plentiful nonrenewable resource, and water contamination has recently risen to the top of the global priority list [122]. Industrial growth, different human activities, the demand for more food, and the indiscriminate use of synthetic agrochemicals all increase contaminants, especially heavy metals (HMs) and pesticides, in aquatic ecosystem components, such as groundwater aquifers [123]. The most common pollutants in agricultural water are pesticides and HMs, which enter the human food chain by entering plant tissues and animal bodies, catastrophically affecting sustainable agriculture and endangering human health. Although some plants have detoxification mechanisms, most do not; consequently, HMs are transported into plants through contaminated water and incorporated into the food chain [98,124]. Some chemical pesticides and several HMs, such as zinc (Zn), copper (Cu), nickel (Ni), cobalt (Co), mercury (Hg), arsenic (As), and lead (Pb), pose strong carcinogenic risks [6]. However, as highly mobile elements, lead (Pb), cadmium (Cd), arsenic (As), and mercury (Hg) are the most toxic [125].

Accordingly, exploring methods for cleaning up HMs and pesticides in environmental components, such as agricultural water, is a research emphasis for sustainable agriculture. Activated carbon, as an effective absorbant, is commonly employed in industry to filter out unwanted substances, such as chemicals and heavy metals, from water systems. Activated carbon is effective in treating wastewater, but it has a high price, so its use is not logical for purifying agricultural water. Different hydrogels have the ability to provide abundant adsorption sites such as -OH, -NH_2_, -COOH, and -SO_3_H, which are useful for binding to target metal ions. CMC is used as a hydrogel with hydroxyl and carboxyl groups as an adsorbent to remove metal ions. Moreover, the high swelling capacity of different water hydrogels helps release heavy metal ions in their networks. The potential of CMC as an active material has been demonstrated in a wide range of water treatment systems [57]. CMC’s hydroxyl and carboxymethyl groups can form chelates with heavy metal ions [126].

**Figure 8:**
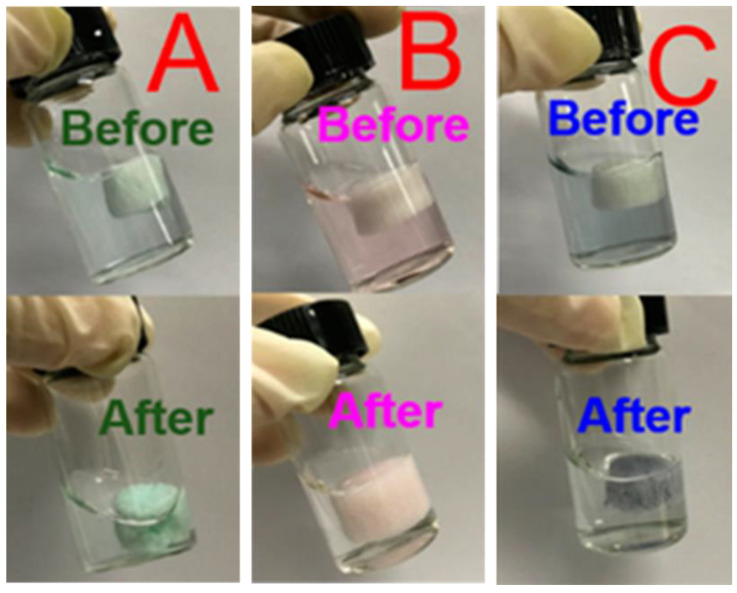
The potential of the prepared absorbers produced based on a metal-organic framework modified by ethylenediaminetetraacetic acid into cellulose nanofiber (CNF) and treated with carboxymethyl cellulose. (**A**–**C**) depict the prepared absorbers filters’ performance in removing Cu^2+^, Co^2+^, and Cr^3+^, respectively [130].

For instance, Cao et al. [127] synthesized monodispersed CMC-stabilized Fe-Cu bimetal NPs that could dechlorinate 1,2,4-trichlorobenzene with 90% removal efficiency. Al Othman et al. [128] synthesized copper-CMC NPs and these NPs could remove tetracycline antibiotics with a 90% removal efficiency from water. The efficiency of chitosan/CMC hybrid adsorbents for the removal of different HMs from wastewater was assessed by Manzoor et al. [129], who showed that these composites, fabricated using arginine cross-linkers, have a great capacity for removing lead and cadmium ions from water. In another practical study, Li et al. [130] prepared an absorbent based on a metal-organic framework modified by ethylenediaminetetraacetic acid (UiO-66-EDTA) into cellulose nanofibers (CNFs). The pore structure of the produced absorbents was modulated using different CMC concentrations. The produced UiO-66-EDTA/CNF/CMC exhibited great potential for the absorption of HMs, with a removal efficiency of 91%. Figure 8 depicts the efficacy UiO-66-EDTA/CNF/CMC absorbents in the absorption of three HMs.

Similar positive results from numerous other studies indicate that CMC-based composite materials can be considered potential substances for removing pesticides and HMs from contaminated agricultural water. Embedding CMC-based absorbents in irrigation equipment or the production of CMC-based filters can effectively bring healthy water to plants and the food chain. In addition to the above capacity, CMC can be used in hydrogel form to absorb pesticides and HMs. Godiya et al. [126] applied hydrogel composites consisting of CMC incorporating polyacrylamide to absorb Cu^2+^, Pb^2+^, and cd^2+^. Similarly, Wu et al. [131] obtained magnetic composite hydrogels to absorb Mn^2+^, Pb^2+^, and Cu^2+^. Composite hydrogels comprised of CMC and alginate incorporating Fe_3_O_4_ nanoparticles showed a great absorption capacity for Mn^2+^, Pb^2+^, and Cu^2+^ by 71.83, 89.49, and 105.93 mg.g^−1^. Although there are few documents regarding the application of CMC hydrogels to absorb pesticides, some documents prove the capacity of CMC hydrogels to absorb pesticides. In this regard, Abdel Gaffar et al. [132] prepared CMC-based hydrogels that have significant potential to absorb 4-chlorophenol and 2,4-D from water solutions. Table 6 shows some studies on the application of CMC to remove heavy metals.

## 5. CMC Applications in Food Industry

To protect food products from microorganisms and physiological activities, stabilizers are needed. Stabilizers are responsible for maintaining physical stability and sensory properties. Stabilizers are used to prevent physical disintegration, stop phase separation in multicomponent formulations, and result in a favorable product taste and odor. Materials used as stabilizers in the dairy industry are polysaccharides and gelatin. CMC is one of the types of polysaccharides that is used as a stabilizer [138]. The ability to form a semipermeable shield against gases and water vapor, help to maintain cell strength, add gloss to coated foods, increase mechanical handling properties, and loss of volatile compounds are other benefits of food coatings [139].

The degree of carboxymethylation for CMC formulated for food applications should be between 0.5 and 1. In other words, it is the degree of substitution and degree of polymerization that determine the performance of CMC in food products. For example, depending on the type of formulation, CMC forms complexes with milk proteins. It is also used in ice cream to control ice crystal growth and in yogurt as a rheological modifier [138].

CMC has been reported to have a wide range of benefits, such as increased viscoelasticity, oil/grease resistance, and enhanced coating properties. Gel-CMC biopolymer composites can form transparent edible coatings or films and act as a carrier for active additives such as antioxidants and antimicrobial agents [36,140].

Maintaining safety and improving nutritional and sensory quality are the most important challenges in food preservation. The shelf life of food depends on many factors, including the quality of raw materials, product formulation, type of preparation, packaging, and storage conditions [141]. Hydrolysis and oxidation of proteins and lipids, enzymatic degradation (texture and color change), and browning are among the events that negatively affect the quality of food products [141,142,143]. Naturally derived biomaterials such as CMC, in the application of other technologies such as nanoencapsulation, extrusion, and emulsion, can delay the deterioration of food compounds. The encapsulation method is used to preserve the nutritional value, taste, and smell and improve the characteristics of biologically active substances [141].

Furthermore, layer-by-layer self-assembly, emulsion techniques, mixing and stirring method, and extrusion-drop method are techniques that increase CMC efficiency [89,144]. In the following, we discuss the applications of CMC as an edible coating for various purposes.

## 6. CMC as Edible Coating Substances in the Preservation of Agricultural Products

Vegetables and fruits have a short shelf life after harvest as a consequence of physiological and biochemical deterioration and microbial decay. Postharvest crop losses can be attributed to several factors, including improper storage and protection, improper packaging, and bacterial and fungal diseases. The high nutritional and water contents of vegetables and fruits make them susceptible to changes in physiological processes, including respiration and metabolism [145]. Overall, this damage reduces the quantities of quality items available to consumers who desire fresh, healthful packaged fruits and vegetables. Therefore, postharvest procedures are essential to reduce the amount of food waste and maximize the marketability of vegetables and fruits. Several physical (e.g., UV light, chilling, heating, freezing, and controlled atmosphere packaging) and chemical (e.g., H_2_O_2_, ozone, bromine, synthetic wax, and chemical fungicide treatments) methods are the most common techniques for the postharvest preservation of fruits and vegetables [146]. However, these techniques all have disadvantages; for example, chilling injury in freezing, vitamin losses in heating, costly applications in packaging under controlled atmospheres, while chemical methods can have negative effects on consumer health and promote environmental degradation [147,148,149,150]. Therefore, any employed strategy must protect the product from postharvest damage and losses while also being economical, safe, ecologically friendly, and straightforward.

An edible coating can be an efficient and eco-friendly choice for increasing storability and diminishing postharvest losses. CMC, as a cellulose derivative that dissolves in water, has been utilized as an edible coating on vegetables and fruits. It is odorless, tasteless, and noncaloric and has excellent layer-forming characteristics that make it useful as a coating or for packing postharvest products. It can allow for longer fresh food storage after harvest by acting as a barrier to moisture and preventing spoilage [140,151,152]. Coating postharvest crops using edible polymers, such as CMC, is a more cost-effective way to package products and results in a significant economic benefit.

CMC has the potential to prevent physical, physiological, and microbial damage to postharvest products [140]. In this regard, the application of active coatings provides significant advantages over common coating layers [153]. An active coating can provide different substances, such as biological control agents (BCAs), NPs, antibrowning agents, antioxidation agents, or other functional materials, to the CMC polymer to generate edible film coatings with antimicrobial, antibrowning, and antisenescence properties that can be applied in many food and agricultural situations. Table 7 shows some applications of CMC as an edible coating in the protection of food products.

### 6.1. CMC-Based Active Coating for Physical and Physiological Protection

**Figure 9:**
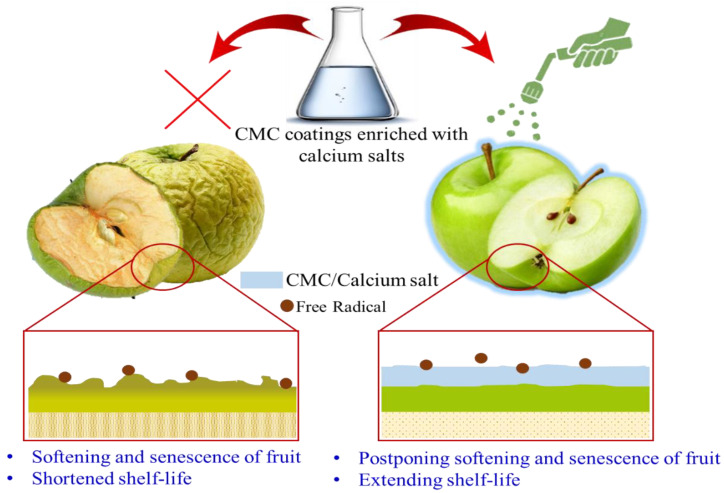
The use of carboxymethyl cellulose (CMC)-based coatings to protect fruit from physical and physiological damage.

The postharvest and during-harvest processing of agricultural products may hurt fruits and vegetables. The physical damage and resulting wounds can lead to the loss of intracellular water and shriveling of fresh tissues, leading to losses in fruit marketability and desirability. Applied edible films, such as CMC, act as physical barriers, boost mechanical strength, and reduce the physiological processes (e.g., transpiration and respiration) that cause the plant material to lose its freshness. Combining CMC with other additive substances can provide a formulation with more potential to preserve the product quality. The formulations that offer more advantages are the active coatings [153]. Several materials, such as CaCl_2_, CaCO_3_, and calcium pectate, have been reported to prevent fruit softening, postpone senescence, and increase shelf life [162]. Figure 9 shows a schematic picture of edible CMC/calcium salt coatings used to prevent fruit surface oxidation, softening, and senescence. Deepthi et al. [163] investigated the effect of calcium salts on the storage behavior of guava fruits and verified that calcium delays ripening and increases fruit shelf life. Moradinezhad et al. [164] assessed the Ca salt effect on increasing the long shelf life of jujube fruits, which have a short shelf life at room temperature. They asserted that the immersion of the fruits significantly delayed fruit rot and shrinking. Applying CMC coatings enriched with some additive agents to strengthen the fruit peel prolongs the shelf life of fruit and vegetables, effectively preserving fruit during the packaging and transportation processes. This approach increases the marketability of damage-susceptible and thin-skinned fruit. An experiment by Alharaty et al. [165] revealed that coatings enriched in CaCl_2_ prolonged the fruit postharvest shelf life for up to 15 days and prevented the establishment of pathogenic fungi on the fruit. Therefore, utilizing CMC-based coatings enriched with calcium salts is a safe way to increase the postharvest shelf life of fruits. Another area of future research is to examine whether these coatings can be used to incorporate calcium into a diet for patients with calcium deficiency or who suffer from osteoporosis.

### 6.2. CMC-Based Active Coating for Microbial Protection

Antimicrobial edible coating films can be practical and safe formulations for extending storage life and reducing postharvest losses. Antimicrobial substances incorporated into edible coating films can obstruct the entrance of phytopathogenic agents, such as bacteria and fungi, and kill phytopathogens. These coatings, by inhibiting the growth and damage caused by microorganisms, can extend the freshness and safety of perishable fruits and vegetables. Among the active ingredients, biological control agents (BCAs; e.g., fluorescent *Pseudomonas* [17,166], *Streptomyces* [149,167], and *Bacillus* antagonistic species [150,168]), plant extracts, biogenic NPs with antimicrobial properties [169,170,171], chitosan biopolymers [172], and essential oils [173] can be added to active coatings to provide antimicrobial potential. The metabolites and bacteriocins produced by antagonistic bacteria reduce the decay of fruits, thereby enhancing the shelf life of postharvest products.

**Figure 10:**
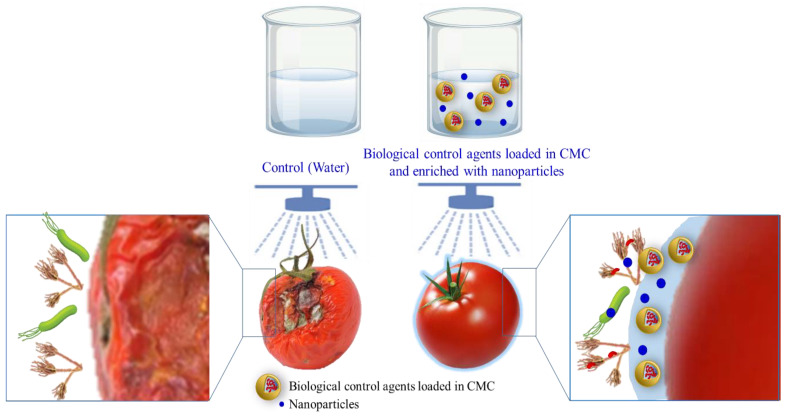
The use of edible active antimicrobial coatings based on carboxymethyl cellulose (CMC) enriched with biological control agents and nanoparticles.

Most BCAs are commercially formulated as wettable powders, liquids, and granular formulations. Desiccation stress, which occurs during the production of a dry formulation, is extremely damaging to living microbial cells and has a profound effect on the viability of active ingredients, particularly nonsporulating bacteria [174,175]. In addition, the efficacy of biological metabolites and agents as free compounds is diminished in liquids and granular formulations due to their volatility, early breakdown, and poor miscibility in aqueous fluids [176]. The application of antimicrobial agents, either BCAs or NPs, directly onto the surface of postharvest products shortens the effective time of the active antimicrobial agents against the target pests or pathogens on the surface of the fruit, increases the need for more dose applications, and reduces business profits. By contrast, loading the agents into edible coatings, such as CMC, maintains an optimum dose of active ingredients on the postharvest surface products. Oliviera et al. showed that *Lipia sidoides* essential oils loaded into CMC had dramatic antifungal effects against *Rhizopus stolonifer* under in vitro and in vivo conditions. Figure 10 shows the effectiveness of edible active CMC coatings enriched with biological control agents and nanoparticles.

Tesfay et al. [159] reported that coating avocado fruits with 1% CMC and moringa leaf extract significantly improved fruit quality and delayed the rate of ripening. Saekow et al. [177] synthesized ZnONPs biologically and assessed the effect of ZnONP-loaded CMC on the quality factors of a tomato and the efficacy of this formula to suppress *Alternaria alternate*. When compared with untreated controls, tomato fruits treated with ZnONPs in a CMC coating showed significantly decreased respiration rates and less weight loss, while the levels of fruit antioxidants increased. They reported that this active formula (CMC+ ZnONPs) delayed the severity of *A. alternate* infection. In a similar study, Yinzhe et al. [178] synthesized an efficient formula by encapsulating brewer’s yeast in CMC and alginate. They claimed that the treatment of grapes with this active coating effectively decreased fruit biomass losses.

### 6.3. CMC-Based Active Coating for Biochemical Protection

In addition to using active coatings as antimicrobial materials, active coatings can be used as a barrier to halt biochemical changes, such as browning and oxidation. Browning is a prominent oxidative reaction that has a detrimental effect on the marketability of fruits and vegetables throughout storage and marketing [179]. In this regard, applying functional coatings with the capability to encapsulate antioxidants and antibrowning agents is a viable strategy for ensuring the long-term storage of fresh-cut fruit with phenolic compounds and delaying the browning of fruit tissues and peel. During the browning process, phenolic compounds are converted into polyphenolic compounds by polyphenol oxidase (PPO), which produces brownish pigments in the presence of oxygen [140]. Applying an edible active coating based on CMC creates a barrier on the surface of postharvest products, reduces oxygen diffusion, and decreases the activity of PPO enzymes, leading to the delayed browning of the products.

**Figure 11:**
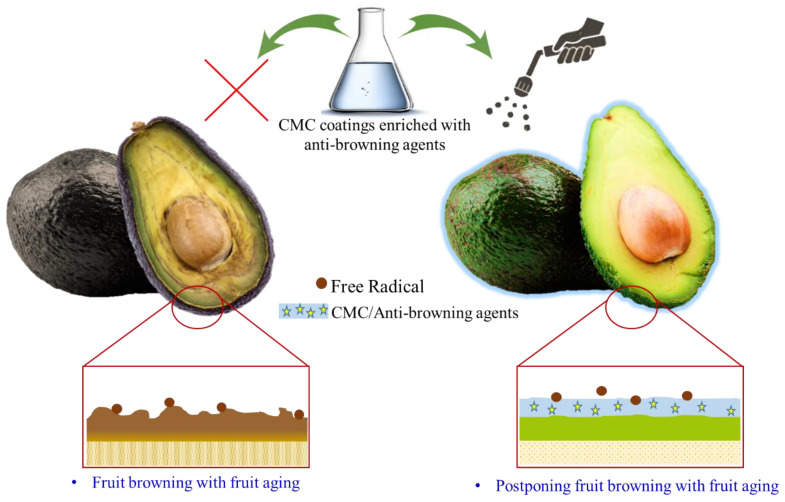
The use of edible active antibrowning coatings based on carboxymethyl cellulose (CMC).

Thivya et al. [180] synthesized active coatings from sodium alginate (SA), CMC, and the wastes from shallot onions (SOWEs). They claimed that the produced film imparted new physical and mechanical features to the coated fruits and had an antioxidant activity. The SA/CMS/SOWEs offered excellent antibrowning activity in fresh-cut potato and apple fruit after 5 and 12 h storage time. Yu et al. [181] enhanced the quality and preservation time of *Brassica chinensis* by coating the vegetables with liquid paraffin containing CMC. The CMC-based coating reduced water loss, improved enzyme activity, and increased the reactive oxidative species (ROS) scavenging activity. Typically, CMC-based coatings provide a barrier on the surface of products and reduce physical lesions and damage while producing phenol compounds, thereby decreasing the browning process. However, an active coating containing antibrowning substances can be produced by adding several ingredients, including ascorbic acid, cysteine, phenolic acids, glutathione, carboxylic acids, resorcinol, and phenolic acids, to the formula. These formulations can increase the marketability of sensitive fruits, such as bananas and avocados, by protecting against oxidative reactions. Using antibrowning agents in fruits with high phenolic compounds is very important and can be effective in increasing the shelf life and marketability of bananas, for example, after harvest. This application is also practical in fresh-cut fruits, such as apples. Figure 11 depicts schematically the application of CMC-based coatings enriched with antibrowning substances.

## 7. Perspectives and Future Outlook 

CMC and CMC-based formulations have recently gained attention due to their edibility, safety, affordability, and ready availability. Future studies on CMC formulations should address the following aspects to inspire future research and increase current knowledge of more recent technologies and CMC-based formulations:

(1) Understanding the sustainability and toxicology issues of the micro- and nano-CMC technologies.

(2) Assessment of the efficiency of superabsorbent hydrogel in retaining water under field conditions.

(3) Enrichment of superabsorbent hydrogels and CMC-based capsules with plant growth-promoting agents.

(4) Synthesis of active coatings enriched with probiotic bacteria that have antimicrobial effects against phytopathogens. Utilizing this type of coating on fruits will decrease the entry of chemical pesticides into the food chain while improving the digestive systems of consumers.

## 8. Conclusions

Increasing global heat, industrialization of countries, drought, soil and water salinity, and soil contamination with heavy metals as abiotic factors have faced many restrictions on agriculture. The emergence of new breeds of pathogens and pests as biotic agents are other limitations of agriculture.

In addition, providing healthy and high-quality food is one of the priorities of governments today. In response to this demand, efforts to produce food have expanded, resulting indirectly in the destruction and contamination of water resources. Indeed, the postharvest coating of vegetables and fruit with nonhealthy materials to extend the shelf life and appearance quality, the entry of pesticides and heavy metals into agricultural water and soil, the destruction of underground water resources, and the use of high doses of agricultural pesticides due to nontargeted application have created a significant gap in sustainable agriculture. Thus, specialists, researchers, and scientists in agriculture should look for solutions to reduce the damages caused by these factors.

The application of alternative materials with multifunctional capacities is important to minimize this gap. Cellulose is the most prevalent biopolymer, as it is present in abundance in the cell walls of plants, algae, and the cell walls of oomycetes. CMC is a cellulose derivative with vast potential for applications in agriculture. As a natural-origin polymer, CMC can participate in delivery systems in various agricultural applications such as chemical fertilizers and lead to the controlled release of chemical fertilizers. In addition, the property of biocompatibility of this material has become a medium that communicates between the plant, the purpose used (irrigation agent, chemical fertilizer, pesticide, fungicide, etc.), and the environment.

CMC is an excellent material for preparing SAHs based on cellulose due to its high water absorbency and swelling ratio in distilled water and salt solutions. Applying this substance to dry soils can be a promising solution for conserving water and increasing irrigation efficiency. However, more study is needed on the swelling kinetics of CMC-based SAHs in various media and on the mechanism by which these hydrogels work. This polymer, with its capability to remove heavy metals, can act as a filter to clean agricultural water and prevent the entry of pollutants into the food chain. Applying this biopolymer to encapsulate BCAs and bioactive metabolites is a novel way to access new formulations of BCAs. In the future, the targeted delivery of biological and chemical pesticides in targeted delivery systems based on edible biopolymers, such as CMC, will reduce the cost of controlling plant phytopathogens. Coating fresh vegetables and fruits with layers enriched with antimicrobial, antibrowning, and antisoftening materials can provide healthy and high-quality products. However, the coating strategies must be meticulously assessed to determine proper techniques for product preservation. Given these optimistic predictions, many opportunities are emerging in each of these fields, supporting the further broadening of the applications of CMC in the future.

## Figures and Tables

**Table 1:** A summary of the application of CMC in agriculture.

Application	Results	Reference
CMC and poly 4-vinylpyridine (P4VP) hy-drogel-N, N, -methylene bis acrylamide	Enhanced the urea absorption	[28]
CMC-PVP hydrogel	Used as potential eco-friendly water-saving materials	[29]
The root targeted delivery vehicle (RTDV) with CMC in wheat	Improvement in seed yield	[30]
Dual-functional redox responsive hydrogel by CMC	Capture heavy metal ions in the soil	[31]
CMC-zein-based nanopesticide delivery system	Improve adhesion and antiultraviolet properties	[20]
Alginate-CMC	Highest encapsulation efficiency; a disease control agent	[32]
NPK fertilizer-CMC- acrylic acid	Easier and faster diffusion of water molecules into the hydrogel, and larger water absorption	[27]
Citric acid cross-linked CMC hydrogels	Control of insects	[33]
CMC is proposed as a coating agent to encapsulate zinc hydroxide nitrate–sodium dodecylsulphate–imidacloprid (ZHN–SDS–IC) for the implementation of controlled release formulation (CRF) in pesticide	Creating an external gel layer on the surface of ZHN-SDS-IC-CMC as an additional barrier that slows IC diffusion.	[34]
Nanoemulsion edible coating using caboxymethyl cellulose	This coating prevented aging caused by oxidative damage of tomatoes by maintaining the level of antioxidant enzymes.	[35]
Avocado peel-coconut-CMC in strawberries	Biopolymer coatings with plant extracts as a potential method for ecological preservation in strawberries against microbial deterioration.	[36]

**Table 2:** Differences between cellulose and carboxymethyl cellulose.

Characteristics	Carboxymethyl Cellulose (CMC)	Cellulose
Solubility in water	Insoluble	Soluble
Mechanical strength	Moderate strength	Moderate strength
Availability	Abundance	Abundance
Sources	This is a derivative of cellulose; however, the synthesis of CMC has been reported from paper sludge, wood residue, textile wastes, mixed office waste, and terry towel waste	Cell wall of plants, algae, and oomycetes
Synthesis method	Alkalization or etherification of cellulose using sodium monochloroacetic acid and different sodium hydroxides	-
Toxicity	Nontoxic	Nontoxic
Applications	As hydrogel, as absorbent, in encapsulation, targeted delivery	In textiles, biomedical, industrial, electronics

**Table 3:** Synthesis of CMC.

Source of CMC	The Method of Synthesis CMC	Characterization Methods	Reference
Mixed office waste	NaOH (0.063–0.156 M); 115 mL isopropanol (30 min at 25 °C); stirring was continued (60 min); predissolved sodium monochloroacetate (0.075–0.118 M) in 10 mL isopropanol; the reaction mixture was heated (40–70 °C) for 1–4 h; alkali with acetic acid (5 M). The reaction mixture was filtered, washed with 70% methanol, and dried at 60 °C in a hot air oven.	FTIR and SEM	[54]
Oil palm empty fruit bunch	NaOH (10–40%); isopropanol (1.5 h to perform the alkalization reaction). The solution was heated at a reaction temperature of 45 to 75 °C for 1 to 4 h (etherification reaction). The slurry was filtered, and the solid product was washed five times with 50 mL of ethanol, followed by a one-time wash with methanol to remove sodium glycolate and chloride, and then dried in the oven at 60 °C for 3 h.	FTIR, XRD, SEM	[61]
Terry towel waste	An amount of 40% NaOH, isopropyl alcohol (alkalization reaction, 90 min); monochloroacetic acid was added to the mixture for 30 min (then kept at 55 °C for 3.5 h). Methanol (70%, *v*/*v*) was added to the reactor, and the mixture was neutralized with acetic acid (90% *v*/*v*). The CMC was recovered by filtration and washed six times with ethanol:water (70:30, *v*/*v*). Finally, the product was washed with methanol and oven dried at 60 °C. Two etherification treatments were performed. The synthesized CMC was then ground and filtered through a 60-mesh nylon cloth.	FTIR, TGA	[55]
Wheat straw	Ethanol, NaOH (for alkalization treatment 1 h at 30 °C) and sodium monochloroacetate (40 °C for 0.5 h); then, the reaction mixture was heated at 70 °C for 2 h. The mixture was cooled to room temperature, added to 100 mL of 80% (*v*/*v*) ethanol, and neutralized with acetic acid. After filtration, the product was washed three times with 80% (*v*/*v*) ethanol and dried in an oven at 50 °C for 16 h.	FTIR, XRD, SEM	[70]
Thai rice straw	Isopropanol and NaOH (for alkalinization process overnight). The methylation process was initiated by adding sodium monochloroacetate to the suspension within 30 min; the reaction mixture was incubated at 50 °C for 3 h. The obtained CMC was purified by suspending it in 70% ethanol and neutralizing the suspension with glacial acetic acid. The CMC was washed with 70% ethanol, 80% methanol, and 95% ethanol. The CMC was dried (vacuum oven at 70 °C overnight).	FTIR, XRD	[71]
Corn husk	NaOH was added to a pure cellulose and ethanol solution (mechanical stirring, at room temperature, 4 h) for the alkalization reaction. The carboxymethylation reaction: monochloroacetic acid (MCA) was slowly added with constant stirring. The product was then filtered and suspended in 200 mL of methanol. The slurry was neutralized using glacial acetic acid. The sample was washed using a 70% ethanol solution and then dried at 60 °C.	FTIR, XRD	[72]

**Table 4:** Application of CMC for encapsulation of bioactive materials.

Microcapsule/Hydrogel	Goal	Result	Reference
QUE ^1^-loaded CHC-CMC nanoparticles	Food Industries	The enclosure of QUE in CDNPs improved its chemical stability and solubility, and higher biological activity.	[100]
Pea proteins-CMC encapsulation of linoleic acid	Food Industries	Better physico-chemical properties.	[101]
RPH ^2^–CMC nanoparticles	Food, Medical	A good biocompatible inhibitor of proliferation of breast cancer cells.	[102]
SAP ^2^-AM ^3^- CMC- -MBA ^4^- loaded with potassium nitrate	Agriculture	The swelling ratio was 190 g/g of dry gel; the amount of released KNO_3_ increased with an increasing loading percentage of SAP.	[103]
PAAm ^5^-MC ^6^-MMt ^7^ loaded with urea	Agriculture	For application in agriculture as a nutrient carrier vehicle.	[104]
Citric acid cross-linked CMC hydrogels and their bentonite composite	Agriculture	Useful for the efficient control of insects having an alkaline gut pH.	[33]
The encapsulation of Bti ^8^ in a matrix of CMC as the polymeric matrix and aluminum sulfate as the gelation agent	Agriculture	In total, 100% mosquito larval mortality, from the second day of treatment, and higher larvicidal activity of Bti at higher temperatures up to 50 °C compared to a nonencapsulated Bti spore/crystal mixture.	[105]

^1^ quercetin; ^2^ superabsorbent polymer; ^3^ acrylamide; ^4^ N,N′-methylenebisacrylamide; ^5^ polyacrylamide; ^6^ methylcellulose; ^7^ calcic montmorillonite; ^8^ *Bacillus thuringiensis* subspecies *israelensis*.

**Table 5:** Application of CMC as a superabsorbent hydrogel.

Method/Goal	Result	Reference
CMC-polyvinylpyrrolidone cross-linked with gamma irradiation and loading urea on hydrogel	Slow urea release, good water retention capacity, being economical, and environmentally friendly	[81]
Superabsorbent hydrogels polyvinylpyrrolidone-CMC of different copolymer compositions by gamma radiation and loading NPK fertilizer on hydrogel	Slow release, high swelling, and slow water retention	[118]
Superabsorbent hydrogels based on cross-linked CMC- acrylamide	As water-managing materials for agriculture and horticulture in drought conditions	[111]
Application of polyvinyl Alcohol-CMC hydrogel as a superabsorbent compound in the soil	Increase water retention in desert regions	[119]
CMC and poly vinyl pyrrolidone synthesized by gamma radiation and loading urea on hydrogel	Slow urea release and good water retention capacity	[81]
Synthesis carboxymethyl cellulose (CMC) via a free radical polymerization technique with acrylamide and 2-Acrylamido-2-methylpropanesulfonic acid (AMPS) as hydrophilic monomers	Nutrient carrier and amendment for sandy soil for advanced agricultural applications	[28]
The copolymer of CMC and mixtures of different comonomers	Suitable in agriculture purposes	[120]
Carboxymethyl cellulose/nano-CaCO_3_ composite amended in the loamy sand soil on maize growth	As an alternative soil amendment for agricultural applications	[121]

**Table 6:** Remediation and heavy metals removal by CMC.

Method/Goal	Results	Reference
Carboxymethyl cellulose (CMC) bridged chlorapatite for removal of zinc and cadmium from water	High uptake of heavy metal from water	[133]
CMC-polyacrylamide for the wastewater remediation	Wastewater treatment and catalytic application	[126]
Nanoparticles stabilized with CMC for in situ destructions of chlorinated ethane	The biological degradation with CMC as the carbon source and hydrogen from the abiotic/biotic processes	[134]
Iron nanoparticles stabilized by (NaCMC) for chromium removal	CMC as an effective stabilizer in nanoparticles for the effective removal of chromium	[135]
A novel biochar supported nanoscale zero-valent iron stabilized by CMC for the removal of chromium	A low-cost, “green”, and effective sorbent for removal of Cr(VI) in the environment.	[136]
Synthesize cross-linked beads from chitosan and CMC with arginine as a cross-linker for adsorption of Pb(II) and Cd(II)	Remove Pb(II) and Cd(II) from aqueous solution with high removal efficiency	[129]
A novel carboxymethyl cellulose sodium (CMC-Na) encapsulated phosphorus (P)-enriched biochar for Pb(II), Cd(II), and Ni(II) removal	A low-cost and high-efficiency adsorbent	[137]

**Table 7:** CMC edible coating in preservation of agricultural products.

Method/Goal	Results	Reference
Polysaccharides from *Osmunda* japonica-CMC (0.7%) for preserve tomato	Increased quality of postharvest tomatoes and reduced weight loss and ascorbic acid	[154]
Locust bean gum/carboxycellulose nanocrystal (LBG/C-CNC) coating for improving properties in strawberries	Antibacterial properties and as effective preservation	[155]
CMC as an edible coating in fresh-cut melons	A superior antimicrobial protection and increased product storability	[156]
CMC extracted from Brewer’s spent grain as a new approach to coating strawberries	Protective properties in room temperature	[157]
Application of CMC with the aim of the development of bio-based films and with new functionalities in coffee grounds	Preservation in the physicochemical properties	[158]
CMC-moringa leaf and seed as a novel postharvest treatment in avocado fruit	Suppressing diseases, prolonging the shelf life, and increase in avocado quality	[159]
The ability of carboxymethylcellulose (CMC)-Astragalus honey (Astragalus gossypinus) to control rancidity and microbial spoilage of pistachio kernel during storage at room temperature	Increase in the shelf life of pistachio kernel	[160]
The effects of CMC on quality aspects of white asparagus	Increase quality of asparagus (with retarding moisture loss and reducing hardening in their basal part)	[161]

## Data Availability

Not applicable.
